# Phenolic Compounds of Grape Pomace Skin Released During SHIME Colonic Fermentation Shape the Transcriptomic Profile of Tight Junctions, Improving the Barrier Properties in Caco-2 Cells

**DOI:** 10.3390/ijms27094123

**Published:** 2026-05-05

**Authors:** Miltha Hidalgo, Francisca Vera, Alina Concepción-Alvarez, Vanessa Rubio, Bárbara Railef, Jorge Meneses-Pacheco, Macarena Moreno, Martina Oyarzún, Adriano Costa de Camargo, Raquel Bridi, Karen Fuenzalida, Elva Gonzales, Igor Pacheco, Carolina Añazco, Rodrigo Pulgar, Omar Porras

**Affiliations:** 1 Laboratorio de Investigación en Nutrición Funcional, Instituto de Nutrición y Tecnología de los Alimentos (INTA), Universidad de Chile, El Líbano 5524, Santiago 7830490, Chile; a24ca90@gmail.com (A.C.-A.); vanessarubiof@gmail.com (V.R.); barbara.railef@gmail.com (B.R.); jorge.meneses@aya.yale.edu (J.M.-P.); macarena.moreno@inta.uchile.cl (M.M.); oyarzunlmartina@gmail.com (M.O.); 2 Laboratorio de Genómica y Genética de Interacciones Biológicas (LG2IB), Instituto de Nutrición y Tecnología de los Alimentos (INTA), Universidad de Chile, El Líbano 5524, Santiago 7830490, Chile; francisca.vera.t@ug.uchile.cl (F.V.); rpulgar@inta.uchile.cl (R.P.); 3 Instituto de Nutrición y Tecnología de los Alimentos (INTA), Universidad de Chile, El Líbano 5524, Santiago 7830490, Chile; adrianodecamargo@inta.uchile.cl; 4 Instituto de Ciencias Aplicadas, Universidad Autónoma de Chile, Santiago 7500910, Chile; 5 Department of Pharmacological and Toxicological Chemistry, Faculty of Chemical and Pharmaceutical Sciences, Universidad de Chile, Santiago 8380000, Chile; raquelbridi@ciq.uchile.cl; 6 Laboratorio de Enfermedades Metabólicas, Instituto de Nutrición y Tecnología de los Alimentos (INTA), Universidad de Chile, El Líbano 5524, Santiago 7830490, Chile; kfuenzalida@inta.uchile.cl; 7 Laboratorio Nutribreeding, Instituto de Nutrición y Tecnología de los Alimentos (INTA), Universidad de Chile, El Líbano 5524, Santiago 7830490, Chile; elvagnutricion@gmail.com (E.G.); igor.pacheco@inta.uchile.cl (I.P.); 8 Laboratorio de Bioquímica Nutricional, Escuela de Nutrición y Dietética, Carrera de Nutrición y Dietética, Facultad de Ciencias de la Rehabilitación y Calidad de Vida, Universidad San Sebastián, General Lagos #1190, Valdivia 5110773, Chile; carolina.anazco@uss.cl

**Keywords:** phenolic compounds, human colonic fermentation, SCFA, TEER, Caco-2, RNA-seq

## Abstract

The association between dietary fiber and phenolic compounds allows the latter to reach the colon, where most polysaccharides undergo fermentation. This bioprocessing weakens the matrix and promotes the release of the phenolic compounds, which then exert beneficial effects on intestinal function. Although this notion is widely accepted, supporting evidence remains scarce. In this study, we subjected grape pomace skin to in vitro digestion to obtain an indigestible fraction suitable for SHIME bioreactors. Throughout these stages, we observed a sequential increase in the release of phenolic compounds, with colonic fermentation playing an important role. Although we did not observe an increase in short-chain fatty acid (SCFA) production by the gut microbiota, we performed a repeated-challenge design on differentiated Caco-2 monolayers. With this approach, we found that the phenolic-rich ferment prevented the transepithelial electrical resistance (TEER) drop on the second challenge and modulated the transcriptomic profile assessed by RNA-seq. Our findings indicate that the Caco-2 cellular responses mentioned above were SCFA-independent and likely due to the differential impact of phenolic compound load after colonic fermentation of grape pomace skin.

## 1. Introduction

Plant-based foods are an excellent source of dietary fiber, which works as an intricate molecular network composed of lignin and the most abundant polysaccharides, such as cellulose, pectin, and hemicelluloses [1], in which phenolic compounds are bound. Among the forces that maintain this molecular relationship between phenolic compounds and biopolymers are hydrophobic interactions, hydrogen bonds, van der Waals forces, and, in some cases, covalent bonds (extensively reviewed by McNeil et al.) [1,2]. In particular, the skin of red grapes serves as a reservoir for a vast array of volatile and phenolic compounds, which are of interest to the wine, juice, and grape derivative industries [3].

More than 100 phenolic compounds have been identified in grape skin by combining liquid chromatography/electrospray ionization with time-of-flight or tandem mass spectrometry [4]. This phenolic-enriched matrix is characterized by resistance to digestion and, therefore, has the potential to drag an important number of phytochemicals to the colonic niche [5]. Once the phenolic-enriched matrix arrives in the colonic lumen, the release of phenolic compounds can be facilitated by the breakdown of these biopolymers exerted by the colonic microbiota [6] or by the specific cleavage of ester/ether bonds between phenolic acids and the polysaccharide chain, as demonstrated for hydroxycinnamic and hydroxybenzoic acids [7]. This second kind of release also applies to proanthocyanidins, also known as condensed tannins, which consist of oligomers and polymers of flavan-3-ol and flavan-3, 4-diols [8]. The combined enzymatic action of the gut microbiota allows phenolic compounds to interact positively with the colonic epithelium. This process provides some support for the hypothesis that bioactive compounds carried by some dietary fibers reduce the risk of inflammatory bowel diseases [9], including colon cancer [10,11,12]. In addition to chronic intestinal illnesses, evidence obtained from animal models supports a protective effect of polyphenolic-rich fiber intake on experimental colitis in rats [13] and mice [14,15].

Most studies have evaluated the biological impact of polyphenolic fractions bound to the anatomical structure of grape skin [16], using alcoholic extracts from this matrix in human intestinal cell models [17,18,19]. Among those studies, only a few have pretreated this food matrix with a simulated digestion process covering the oral, gastric and intestinal stages [19,20] and even fewer have continued with further in vitro colonic fermentation, as reported recently by Wang et al. [21]. In that study, the release of polyphenolic compounds from three varieties of grape pomaces was evaluated at 6, 12, and 24 h, demonstrating the capacity of the human gut microbiota to release phenolic compounds, which, in turn, modify the relative abundance of microbial community members and promote SCFA production [21]. However, the direct impact of the fermented polyphenolic-bound fraction present in grape pomace on colonic epithelial cells has not been evaluated yet.

In this study, we processed grape pomace skin by in vitro digestion followed by simulated human colonic fermentation in SHIME^®^ bioreactors. This approach allowed for the characterization of phenolic content and SCFA levels, while also providing a realistic ferment suitable for apical exposure in differentiated Caco-2 cell cultures. Upon differentiation, cell-to-cell contacts become so tight that ion flux through the monolayer is limited, enabling non-invasive monitoring of transepithelial electrical resistance [22]. By doing this, we observed that ferment rich in phenolic compounds improved barrier function in this cellular model, an effect not attributed to SCFAs. To expand on this observation, we performed RNA sequencing on the treated cells, which unveiled a higher expression of genes related to tight junctions, thus further explaining the enhanced barrier integrity induced by the polyphenolic-bound fraction. These data, along with changes seen in the mRNA expression profile at the apical plasma, suggest that phenolic compounds released during colonic fermentation by the microbiota have an SCFA-independent biological impact on intestinal epithelia.

## 2. Results

### Release of Phenolic Compounds from the Grape Pomace Skin by In Vitro Gastrointestinal Digestion

The skin from grape pomace derived from Carménère berries was subjected to in vitro digestion, allowing separation of the digested material by centrifugation, which is denoted here as the non-digestible fraction (see Table 1). The phenolic compounds present in both fractions obtained during the gastric and intestinal steps were extracted using a methanol:acetone mixture. With this method, syringic and synapic acids were the only two phenolic acids present in the skin pomace before digestion, which were not detected during gastric digestion, either in the digestible or the non-digestible fractions. However, gastric digestion weakens the matrix since some flavonoids, such as kaempferol and taxifolin, become extractable from the non-digestible fraction. Further intestinal digestion, in which pancreatin is added to the non-digestible fractions obtained in the previous step, allowed the detection of abscisic acid along with the above-mentioned phenolic acids, including the detection of syringic acid in both fractions. Similar to the effect of gastric digestion on the matrix, the intestinal processing allowed the extraction of more kaempferol and quercetin from the non-digestible fraction, including the flavan-3-ol epigallocatechin gallate.

**Figure 1:**
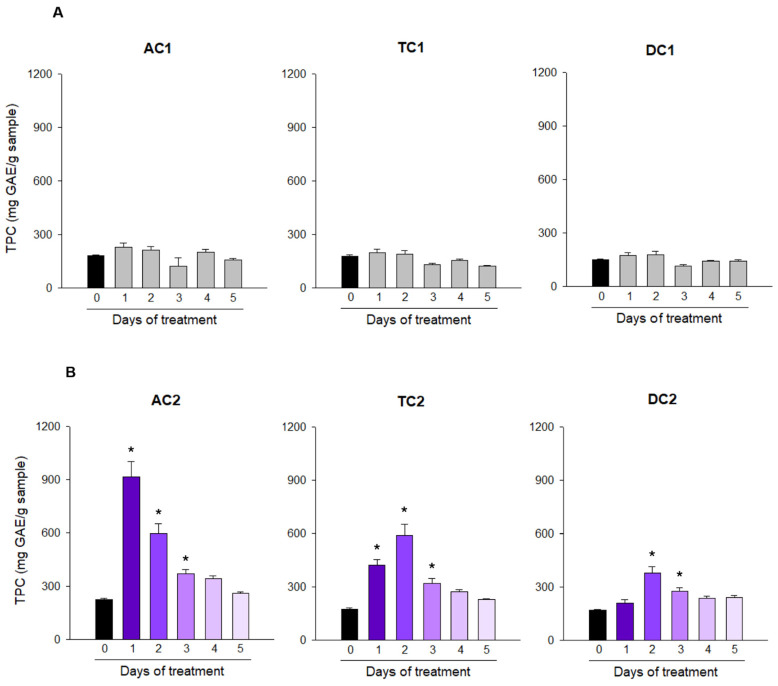
Time course of total phenolic content (TPC) determined in the ascending (AC), transverse (TC), and descending colon (DC) of TWINSHIME after adding the indigestible fraction of grape pomace skin treatment. The indigestible fraction obtained after in vitro digestion of grape pomace skin was introduced at the stage corresponding to the small intestine of each line of TWINSHIME. As a control, (**A**), a phenolic-depleted indigestible fraction was used, while the other line, (**B**), it received an intact and equivalent amount of indigestible fraction. The value assigned to Day 0 corresponds to the mean ± SE of the four days preceding the treatment. For the next days, TPC was determined in triplicate using the Folin–Ciocalteu method and expressed as the mean ± SE. Statistically significant differences with respect to day 0 are indicated with an asterisk (*) as measured by one-way repeated-measures ANOVA with post hoc Bonferroni analysis.

This in vitro digestion generated an insoluble and non-digestible fraction that was incorporated in one of the bioreactors corresponding to the ascending colon of the treated SHIME line. As a control, the other line received the same amount of non-digestible fraction, which had been depleted of phenolic content through eight exhaustive extractions of one hour each [two rounds of two methanol, two of acetone, and two of butanol: HCl; and two rounds of 10M NaOH]. This procedure effectively reduced the fiber’s phenolic content, as evidenced by the total phenolic content (TPC) in vessels from the control line, which did not increase (Figure 1A). In contrast, the vessel corresponding to the ascending colon of the treated line that received a non-digestible fraction with the phenolic compounds still trapped in the matrix showed significantly elevated increases in TPC (Figure 1B). As expected, the fluctuation in the TCP levels in the treated line was consistent with the dynamic transfer between the bioreactors, showing a peak on the first day in the ascending colon, followed by the transversal and descending colon that showed their peaks on their TPC level on the second day after treatment, with a lower magnitude than the ascending colon, as expected by the dilution and microbial activity.

After 24 h of treatment, we collected samples from the ascending colon to assess the impact of microbial activity on the release of phenolic compounds from matrices of the incorporated non-digestible fraction. As shown in Table 1, the flavonol, rutin trihydrate, and the flavan-3-ols, catechin and epicatechin, were released during this period of fermentation by human colonic microbiota.

In addition to the increase in TPC and the release of some phenolic compounds at the ascending colon, we wondered if the fermentative capacity of the microbial community increases in the presence of this polyphenol-rich fiber by determining the levels of acetate, propionate, butyrate, and valerate over a five-day treatment period to evaluate this attribute of the microbiota. None of these four short-chain fatty acids increased significantly in the ascending colon or the following vessels corresponding to the transversal and descending segments, indicating that fermentation products did not accumulate at any point in the treatment (see Supplemental Figure S1).

**Figure 2:**
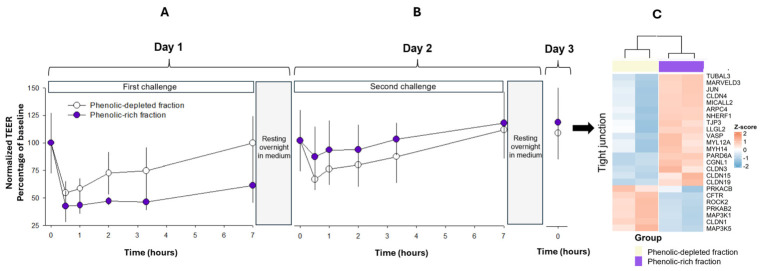
Effect of colonic ferments from the indigestible fraction of grape pomace skin on the transepithelial electrical resistance of Caco-2 cell monolayers. Caco-2 cells were seeded onto bicameral inserts and cultured for 25 days, after which they were exposed to two sequential apical challenges. These challenges were conducted by replacing 50% of the culture medium with the ferment obtained from the ascending colon after 1 day of exposure to either phenol-rich or phenol-depleted fraction. The first challenge lasted 7 h (**A**), and the TEER was measured right before and after 0.5, 1, 1.7, 3.3, and 7 h. Subsequently, the apical medium was replaced with fresh culture medium, and the cells were left undisturbed to recover in the incubator. The following day (**B**), a second challenge was performed under the same conditions. On the third day, TEER was measured again, and RNA isolation was performed for subsequent RNA-seq analysis. In addition, a heatmap was generated from the RNA-seq data to visualize the fold changes in the expression of genes involved in tight junction complexes (**C**). Each TEER value represents the mean ± SEM from 3 biological samples obtained in 2 independent experimental assays. TEER values were normalized to the measurement obtained at time 0, which was used as the baseline, and are expressed as a percentage of the baseline TEER.

Our next step was to expose a monolayer of differentiated Caco-2 cells, a cellular model of the intestinal epithelium, to these colonic fermenta and assess their impact on cellular barrier properties by measuring transepithelial electrical resistance. After twenty-five days growing on Transwell membranes, monolayers of Caco-2 cells exhibited TEER values over 700 Ω*cm^2^ [711 ± 130, *n* = 9] (see Supplemental Figure S2). Under this condition, Caco-2 cells were exposed to the SHIME ferment obtained from the ascending colonic vessels with the phenolic-rich fiber or its phenolic-depleted counterpart. As shown in Figure 2A, we could observe a decrease in the electrical resistance of the cell monolayer within the first 30 min of exposure to the colonic ferments. This phenomenon likely results from the complex nature of colonic fermentation, which, despite filtration, may still contain endotoxins, flagellins, and other bacterial components that are potentially harmful to epithelial cells. However, after this initial decrease in TEER values, a sustained recovery phase followed rather than the progressive decline that was expected. This finding motivated us to keep the cells alive to observe if the TEER values fully recovered. Surprisingly, the TEER values in the group of monolayers exposed to the ferment with the phenolic-depleted fiber were 1080 ± 298 Ω*cm^2^, as well as in those exposed to the ferment with the phenolic-rich fiber, which reached 780 ± 225 Ω*cm^2^, indicating a total recovery of barrier integrity in 24 h. Given the opportunity, we performed a second exposure on these monolayers; this time, we observed a drop in the TEER only in the group of cells exposed to the ferment with the phenolic-depleted fiber. On the contrary, those monolayers of Caco-2 cells that received ferments with the phenolic-rich fiber were unaffected (see the second challenge in Figure 2B).

This experiment revealed that differentiated Caco-2 cells are a robust model that withstands 7 h of apical exposure to colonic fermentation and then completely recovers within 24 h. Moreover, the second exposure to these two classes of ferments helps us to visualize a sort of adaptation of the monolayers that were exposed to fermentations generated with phenolic-rich fiber. Therefore, we decided to determine whether this cellular adaptation was a product of a differential transcriptional profile that explains the resistance of the epithelial monolayer by performing massive sequencing of messenger RNAs in these two groups of differentiated Caco-2 cells. It is worth mentioning that principal component analysis of the dataset indicated that data from treated cells with ferments of phenolic-rich fiber clustered together, being separated from cells under the control condition (see Supplemental Figure S3).

**Figure 3:**
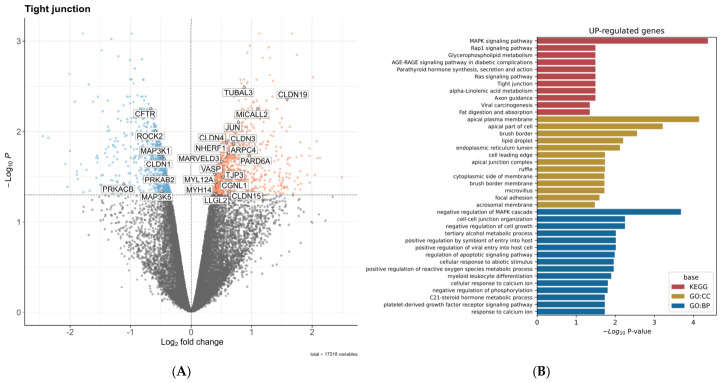
Transcriptomic response of Caco-2 cell monolayers to a phenolic-rich colonic ferment. Caco-2 cells were cultured for 25 days and subjected to two successive challenges with grape pomace colonic ferment as described above. After completion of the second challenge, and following TEER verification 24 h after medium renewal at the 7 h timepoint, total RNA was extracted for RNA-seq analysis. (**A**) Volcano plot showing RNA-seq gene expression changes in tight junction-related genes. (**B**) Functional classification of differentially expressed genes based on Gene Ontology Cellular Component (GO:CC) and Gene Ontology Biological Process (GO:BP), and pathway enrichment according to the Kyoto Encyclopedia of Genes and Genomes (KEGG).

In order to visualize which genes experienced changes in their expression after repeated exposure to phenolic-rich fiber ferments, we elaborated a Volcano plot (Figure 3A), in which red dots correspond to statistically significant increases in gene expression, as is the case for the genes involved in tight junction protein signaling, whereas blue dots are the genes whose expression became significantly decreased by the treatment. As can be observed in the heatmap (Figure 2C), among the genes that showed an increased expression, we found Claudins 3, 4, 15, and 19, TJP3, and MarvelD3, which are directly involved in the formation of paracellular channels, although the gene coding for claudin1 elicited lower expression with the treatment. Others, like NHERF-1, which are involved in the transepithelial Na+ and fluid influx, also showed increased expression, whereas the gene that codifies for the chloride ion channel CFTR marked a diminished expression. Some of the genes with increased expression codify for proteins related to cytoskeleton organization, like ARPC4, Myosin Light Chain 12A (MYL12A), MYH14, and GCNL1. We also observed an increased expression in genes involved in maintaining cell differentiation, such as the proto-oncogene JUN or AP-1, Micall2, LLGL2, and TUBA/PARD6. Collectively, all the up-regulated genes that we found are topographically assigned to the apical and membranous cellular domains, according to the ontology gene database for cellular components (OG:CC), and are involved in cell-to-cell junction organization, along with metabolic and regulatory process in proliferation, according to the ontology gene database for biological process (OG:BO) and the Kyoto Encyclopedia of Genes and Genomes (KEGG) (Figure 3B).

## 3. Discussion

In this work, we followed the sequential release of phenolic compounds from the epicarps of Carménère grape pomace through an in vitro simulation of gastric and intestinal phases, including a fermentation step in a realistic colonic environment with human gut microbiota. As expected, an increasing number of phytochemicals were detected in the soluble fraction after each digestive step. Further bioprocessing by the colonic microbiota weakened the matrix present in the insoluble, non-digestible fraction, releasing additional compounds into the ferments. Although we observed an increase in the total phenolic content by more than threefold, it was not accompanied by an increase in the fermentative capacity of the microbial community. This lack of effect allowed us to evaluate the impact of two colonic ferments with different phenolic loads but equivalent SCFA levels on a monolayer of differentiated Caco-2 cells. Both ferments resulted in a transient drop in TEER values, which recovered in a matter of hours. Curiously, a second challenge with the same ferments after a 24-h rest period revealed subtle differences between the monolayers exposed to phenolic-rich ferments and the controls. Although TEER values decreased by a similar magnitude, the monolayers exposed to the phenolic-rich ferment recovered more efficiently than those exposed to the control ferment. This subtle observation was accompanied by a shift in the transcriptomic profile toward genes directly involved in tight junction assembly, cytoskeletal interactions, and ion and fluid transport. The biological impact described cannot be attributed to the well-known fermentation products, the SCFAs; instead, microbiota-released phenolic compounds present in the fiber or microbially derived bioactives promote the up-regulation of genes associated with cell-to-cell adhesion, polarity organization, and the maintenance of differentiated phenotypes.

The polysaccharide structure of the insoluble fraction resulting from in vitro digestion is composed of hemicellulose chains as the major component, which include xyloglucans, mannans, xylans, and arabino-galactans, with specific patterns of substitutions, followed by other components such as cellulose, lignin, and pectin [23,24]. All these biopolymers become cross-linked by phenolic compounds, such as hydroxycinnamates, forming a complex, undigestible network that drags these phenolic compounds to the colonic environment [25,26,27]. Indeed, the fiber content found in epicarps from red grapes is quite high, oscillating between 50 and 75% [28,29] even in the indigestible fraction [8]. Among the phenolic compounds present in the above-mentioned fraction, we identified and quantified syringic, sinapic, and abscisic acids; only syringic acid has been reported to be present as a phenolic acid bound in grape skins [27]. In a recent report from Costa de Camargo’s group, ethanolic extractions of Carménère grape pomace enabled the detection of gallic acid in the range of 300–600 mg/g (dry weight), depending on the ethanol-to-water ratio used [30]. Although the same grape variety was used in this work, different solvent-extraction procedures were applied, rendering similar amounts of syringic acid (471 μg/g), whose chemical structure only differs in the methylation on the hydroxyl groups from gallic acid, indicating perhaps that extraction procedures have a differential impact on the structural integrity of phenolic compounds. Other compounds belonging to the group of flavan-3-ols, such as catechin and epicatechin, appear after the first 24 h of fermentation at 188 and 6 μg/g, respectively. Similar amounts of catechin have been reported in the skin of grapes [31]; however, concentrations over ten times higher have been reported in seeds and stems, the other components of grape pomace, highlighting the importance of specifying how the grape pomace was processed and which part was analyzed.

Other vegetal matrices have been subjected to in vitro digestion, demonstrating that phytochemicals are released continuously across successive digestive stages, leaving a subset of bound phenolic compounds available to interact with the colonic microbiota. In the case of some members of the Gramineae family, in vitro digestion of brans from rice and wheat, which present massive amounts of ferulic acid, showed that upper digestion stages release minimal quantities of phenolics. Furthermore, colonic fermentation has been shown to be far more efficient at releasing ferulic acid than digestion, either when human fecal homogenates [32] or fecal samples from rats fed with wheat bran (1.2 g/Kg) were analyzed [33]. Consistent with these results, sequential in vitro digestion and fermentation of fruits such as *Rosa roxburgii* [34], *Mangifera indica* [35], and *Litchi chinensis* [36] indicate that a subset of compounds, frequently from the cinnamic or hydroxybenzoic acid families, reach the colonic niche [37,38].

Once in the colon, fiber-bound phenolic compounds have the potential to interact with the human gut microbiota and modify short-chain fatty acid production. Reports from Li’s team indicate that insoluble fibers obtained from different varieties of red grape pomace (cabernet sauvignon, merlot, and marselan) induced an increase in propionate levels by approximately two-fold, while leading to a five- to ten-fold decrease in butyrate levels [21]. A similar pattern was found when studying the insoluble fiber from the skin of Pinot noir pomace [39]. In another recent work, no effect on gut fermentation was reported after the incorporation of insoluble fiber from grape pomace into reactors inoculated with human fecal samples using a similar time window as the studies mentioned above [40]. In our case, we did not observe significant changes in SCFA levels in the ascending, transverse, and descending colons of the SHIME system. There are fundamental differences across the studies, such as the fecal sample dilutions used and the load of fiber that enters fermentation, both defining the system’s fermentative capacity and diversity of settings, which could explain the variability in the reported results. Considering these variables, we operated the SHIME reactors at 5% inoculum with a fiber input of 8 mg/mL, which is similar to the settings found in Wang et al. [21], but using a two-fold diluted microbial ecosystem instead. On the other hand, we must consider the capacity of gut microbiota to respond to dietary fiber with an increase in fermentation. It is plausible that the microbial biochemical machinery involved in the sequential breakdown of polysaccharides functions near its maximum rate before any input. For instance, propionigenic, butyrogenic, and acetogenic enzymes, expressed in a subset of individuals from the bacterial community, have the potential to increase their V_max_ by promoting the growth of SCFA-producing members. Indeed, quercetin has been reported as a dietary agent capable of restoring impaired SCFA production induced by ovariectomy in rats [41] and antibiotic-treated mice [42]. Whilst exposing the human gut microbiota to this flavonol for 24 h resulted in a slightly less diverse microbial ecosystem [43], no impact of quercetin on the growth of Bifidobacterium strains was observed within a 30-min to 48-h exposure time window [44]. Similar to quercetin, administration of flavan-3-ol epigallocatechin gallate in the diet resulted in modifications in the composition of the gut microbiota of rats, along with a lower cecum content of butyric and acetic acids [45]. Additionally, in mice subjected to antibiotics and subsequently infected with *Clostridium difficile*, oral administration of 50 mg/kg of flavan-3-ol epigallocatechin gallate restored the intestinal production of SCFAs and improved the barrier properties and inflammatory status [46]. Other members of the flavan-3-ol group, such as catechin and epicatechin, can also modulate the human gut microbiota in anaerobic culture vessels [47]. These observations highlight the challenge of predicting a bacterial community’s response when exposed to a mixture of dietary bioactive compounds. Moreover, bacterial growth is influenced by hierarchically superior factors, such as quorum sensing, which has been well-described in the context of biofilm formation [48,49]. Indeed, there is evidence that fermentation modifies the bioactivity of *Lonicera caerulea* polyphenols, enhancing their capacity to inhibit the formation of dental caries [50]. Thus, quorum-sensing modulation has been raised as another plausible regulatory mechanism that could explain the health effects of polyphenols associated with dietary fiber [51].

Because SCFA levels were comparable between the human colonic bioreactors treated with phenolic-rich and phenolic-depleted indigestible fractions, we were able to evaluate the impact of these ferments on differentiated Caco-2 cells. Specifically, we monitored the evolution of TEER values over two consecutive 7-h challenges. By employing this experimental design, we provided sufficient time for cellular adaptation to be reflected in different patterns of gene expression. TEER values from cells treated with high-phenolic-content colonic ferments changed less compared to those of control cells, which were exposed to ferments with a very low phenolic content, hinting at some sort of robustness to endure this colonic broth, rich in microbial components. Further transcriptomic analyses of these cells revealed significant overexpression of genes involved in tight junction function. The CLDN3, CLDN4, CLDN15, and CLDN19 genes were significantly up-regulated by treatment with ferments rich in phenols. Claudin-3 and -4 isoforms have been previously detected in Caco-2 cells [52,53,54]. In the case of claudin-3, it is overexpressed following several hours of treatment with 100 μM flavonoids, such as kaempferol and pentamethoxyflavone [54,55,56]. Claudin-4 protein levels, by contrast, showed no change upon exposure to kaempferol, but exhibited a slight increase in the presence of the mycotoxin deoxynivalenol [54]. Regulation of claudin-15 and -19 is poorly understood; data from rat intestines and in vitro assays in Caco-2 cells support a role for claudin-15 in Ca^2+^ absorption under physiological conditions when circulating levels of prolactin are higher, such as pregnancy, lactation, and suckling [57]. Interestingly, the expression of claudin-3 and -15 follows opposite dynamics in the presence of sweeteners like aspartame or sucralose. While the sweeteners induced a decrease in superficial claudin-3, a phenomenon dependent on the sweet taste receptor T1R3, the same stimulus induced an increase in superficial claudin-15, which was independent of that receptor [42]. Our results indicate that the repeated exposure of phenolic compounds present in colonic ferments shifted the pattern of claudin expression in Caco-2 cells, characterized by a decrease in claudin-1 and an increase in claudin-3, -4, -15, and -19. It is worth noting that an extracellular motif that recognizes the *Clostridium perfringens* enterotoxin is shared between all the claudins whose expression levels rose [58]. The amino acid consensus sequence of these extracellular segments has been postulated to be critical for stabilizing the β-sheet structure of claudins, which is exposed to the paracellular space and could, therefore, contribute to cell-to-cell adhesion, barrier formation, and channel function of the tight junction complex [59].

It seems that the expression profile of claudins in Caco-2 cells is tightly linked to the specific nature of apical stimuli. For instance, an oxidative challenge (1mM H_2_O_2_, 3h) induces a downregulation of claudin 1 and 3 [60], whereas a 12 h exposure with an inflammatory cocktail (basolateral: TNF-a, IL-1b, and INF-g; apical LPS) leads to higher levels of transcription for claudin 1 and 4. Additionally, apical exposure to isolated phenolic compounds such as ferulic acid (40 mM, 14 h) did not affect claudin-1 expression, but was effective in preventing the loss of barrier integrity induced by sodium arsenate [61]. Moreover, theaflavins (catechin dimers, 20 mM, 3 h) induced a discrete but significant increase in the protein abundance of claudin-1 and 3, a phenomenon related to the AMPK activity [62]. Taken together, these earlier reports and our own results demonstrate that claudin regulation is a highly complex process.

The increased expression of other proteins involved in tight junction formation, such as MARVELD3, supports the hypothesis that colonic fermentation with high phenolic content strengthened the intestinal epithelial barrier. MARVELD3 corresponds to the third member of the tight junction-associated occludin family, which co-localizes with tight junctions, but whose depletion strikingly generates a Caco-2 monolayer with higher TEER values without affecting permeability. These data suggest that MARVELD3 may be a fine regulator of claudin function by affecting their oligomerisation [63,64]. On the other hand, VASP (Ena/vasodilator-stimulated phosphoprotein) and CGNL1 (cingulin) are proteins associated with cytoskeleton dynamics; although they may not be essential for tight junction conformation, they could participate in the organization of the zonula adherens [65,66,67]. Finally, we observed a decrease in the abundance of transcripts for the CFTR ion channel (Cystic Fibrosis Transmembrane Conductance Regulator) along with an increase in mRNAs encoding the Na^+^/H^+^ exchanger regulatory factor NHERF1. The latter interacts through the C-terminal PDZ motifs located in the CFTR protein modulating its intracellular trafficking [68,69] and activity [70], an interaction with relevance for intestinal fluid loss observed in enterotoxin-induced travelers’ diarrhea [71]. Consistent with CFTR downregulation, the phenolic load present in colonic ferments also decreased NKCC1 mRNA, which encodes the Na^+^/K^+^/Cl^−^ cotransporter and whose activation by glucose has been proposed as the mechanism underlying the failure of glucose-based oral rehydrating solutions to reduce stool output [72].

Additionally, we observed increased transcription of SLC2A5, which encodes the fructose transporter GLUT5. This finding contrasts with the report by Zakłos-Szyda et al., in which Caco-2 cells exposed to *Brassica juncea ethanolic extracts* for 36 h induced a decrease in GLUT5 protein levels [73]. We also observed an increase in SCL16A13, which encodes the monocarboxylate transporter 13 (MCT13), likely functioning as a basolateral oligopeptide transporter in Caco-2 cells; this function is enhanced by the loss of electrochemical potential at the plasma membrane and mitochondria [74].

In this study, we simulated the journey of skin grape pomace through the gastrointestinal tract to obtain an indigestible fraction that would ultimately be processed by the colonic microbiota. By monitoring the release of phenolic compounds at each stage, we confirmed that after 24 h of colonic fermentation, several phenolic compounds become available. However, the arrival of the indigestible fraction to the colonic microbiota, regardless of its phenolic content, did not increase SCFA production. Despite this, we demonstrated that the differential load of phenolic compounds, rather than SCFA levels, was sufficient to induce a differential response of electrical conductance of Caco-2 monolayers upon repeated exposure to colonic ferments, as assessed by TEER measurements, and to modify the transcriptomic profiles of these monolayers. Although our data points to which phenolic compounds could potentially reach the colonic niche, the full diversity of metabolites generated by the microbial machinery remains largely uncharacterized. More importantly, it remains to be determined whether these release patterns would stay consistent across different colonic bacterial ecosystems when assessing inter-subject variability, which is a severe limitation of this study. In addition to the metabolic diversity, the temporal dynamics behind the biological effects induced by dietary fiber-associated phenolic compounds, as well as the necessary intake frequency, warrant further research. By integrating the use of more complex models, such as gut organoids, with experimental designs focused on short-time fermentation, we may achieve a deeper understanding of the molecular mechanisms underlying the benefits of phenolic compounds and, thus, provide support for healthy diet recommendations.

## 4. Materials and Methods

### 4.1. Reagents and Standards

The reagents, such as acetone, hydrochloric acid, hexane, sodium hydroxide, ethyl acetate, Folin–Ciocalteu, sodium carbonate, gallic acid, sodium acetate, 2,4,6-tri(2-pyridyl)-s-triazine (TPTZ), ferric chloride, dibasic sodium phosphate, monobasic sodium phosphate, sodium bicarbonate, HPLC-grade methanol, and HPLC-grade acetonitrile, were purchased from Merck (Darmstadt, Germany). The standards for HPLC, including chlorogenic acid, caffeic acid, syringic acid, p-coumaric acid, ferulic acid, rutin, quercetin, and kaempferol, were supplied by Merck (Darmstadt, Germany). Media culture and supplements were purchased from Science Cell Research Laboratories (Carlsbad, CA, USA).

### 4.2. Pomace Sample

Grape pomace of the Carménère variety corresponds to the harvest of 2022 and was cultivated at Viña Acosta, located in the Cachapoal Valley, Fundo El Llano, Lote 6, San Vicente de Taguatagua, O’Higgins Región (34°27′49” S 71°00′33” W). Pomace was directly taken from bins filled with once-pressed grapes two weeks before. Each grape pomace skin was carefully separated by hand from seeds and rachis and immediately stored at −80 °C until processing.

### 4.3. Phenolic Compound Extraction

Skins from grape pomace (0.5 g) were ground in liquid nitrogen to perform an exhaustive extraction consisting of four sequential steps: (i) samples were incubated in a methanol:water (1:1) solution adjusted at pH 2 for 60 min at room temperature, shaking gently at 90 rpm. Then, samples were centrifuged at 2500g for 5 min (Hermle Z 206A, Labortechnik, Germany), and the supernatant was collected (sup-I), and the pellet (p-I) followed a second extraction. (ii) p-I was resuspended in an acetone:water (70:30, pH2) solution following the same procedures described above, obtaining a supernatant (sup-II) and pellet (p-II). (iii) This pellet, p-II, was resuspended in a butanol:HCl (95:5) solution with 0.7 g FeCl_3_ and incubated at 100 °C for one hour of continuous shaking. A supernatant (sup-III) and pellet (p-III) were obtained by centrifugating as described above. Finally, in (iv), the p-III was resuspended in 2.9 M NaOH (8 mL) and incubated for 16 h at room temperature in continuous agitation. By centrifugation, a supernatant (sup-IV) and pellet (p-IV) were obtained. Each supernatant obtained during this procedure was further analyzed by HPLC/DAD.

### 4.4. Gastrointestinal In Vitro Digestion

To simulate the human gastrointestinal digestive process, we followed a standardized protocol of in vitro digestion described in [75]. Skins from grape pomace (2 g) previously pulverized in liquid nitrogen were subjected to oral, gastric, and intestinal stages of digestion. A quick centrifugation step (3000 g for 15 min, 25 °C) was performed to separate the supernatant, the digestible fraction (DF), from the pellet, which is considered the non-digestible fraction (NDF). The supernatants obtained in the gastric and intestinal stages (DF-g and DF-i) were subjected to the first two extraction steps described above. In contrast, the pellets, NDF-gastric (NDF-g) and NDF-Intestinal (NDF-i), were subjected to the complete sequence of four extractions.

For digestion, all stages were performed at 37 °C with orbital shaking at 120 rpm. The oral stage was carried out in a simulated saliva fluid [in mM: 15.1 KCl, 3.7 KH_2_PO_4_, 13.6 NaHCO_3_, 0.15 MgCl_2_·6H_2_O, 0.06 (NH_4_)_2_CO_3_, and 1.5 CaCl_2_·2H_2_O, pH 7] for 2 min. The gastric stage included pepsin (2000 U/mL) in a gastric fluid [in mM: 6.9 KCl, 0.9 KH_2_PO_4_, 47.2 NaCl, 25 NaHCO_3_, 0.12 MgCl_2_·6H_2_O, 0.5 (NH_4_)_2_CO_3_, and 0.15 CaCl_2_·2H_2_O, pH 3] for 2 h. The intestinal stage contained 100 U/mL pancreatin (Cat. No. P7545, Sigma-Aldrich, St. Louis, MO, USA) and 10 mM bile salts (Cat. No. B3883, Sigma-Aldrich, USA) in an intestinal fluid (in mM: 6.8 KCl, 0.8 KH_2_PO_4_, 38.4 NaCl, 85 NaHCO_3_, 0.33 MgCl_2_·6H_2_O, and 0.6 CaCl_2_·2H_2_O, pH 7) for 2 h. Digestion was stopped by rapidly freezing the tubes in liquid nitrogen.

### 4.5. Colonic Fermentation in TWINSHIME^®^

**Figure 4:**
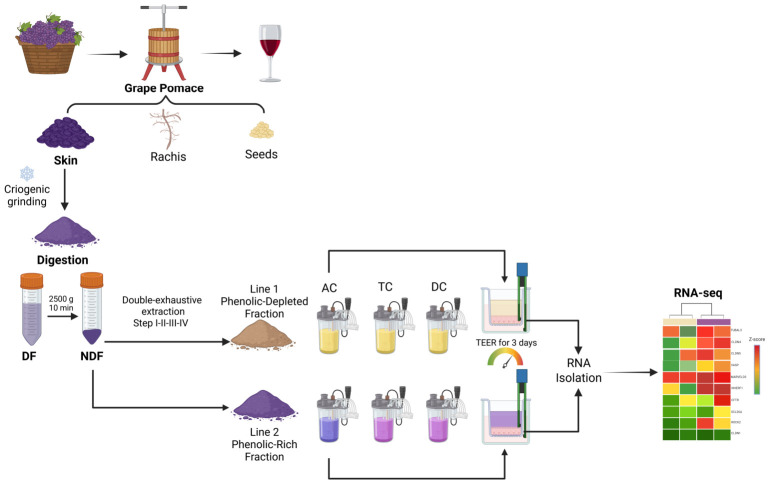
Workflow with grape samples from the harvest to bioassays. Once the grape berries were pressed, the grape skin was manually separated from the pomace, cryogenically milled, and subjected to simulated gastrointestinal digestion to obtain a non-digestible fraction (NDF). This NDF was further depleted of phenolic compounds, denominated as Phenolic-Depleted Fraction (Line 1); the intact NDF was called Phenolic-Rich Fraction (Line 2). Both fractions were incorporated as a single dose to TWINSHIME^®^ colonic bioreactors simulating three colon segments (ascending -AC-, transverse -TC-, and descending -DC-). After 24 h of colonic fermentation, ferments from the AC were applied to an in vitro epithelial model (Caco-2 cells in a bicameral system) to measure transepithelial electrical resistance (TEER) and to analyze the transcriptome by RNA-seq.

The human colonic fermentation was recreated with the TWINSHIME^®^ system (ProDigest, Ghent, Belgium), which allows the simultaneous work of two identical lines, each one composed of five vessels corresponding to: the stomach, the duodenum, and three colonic segments: ascending (AC, pH 5.6–5.9, 500 mL), transverse (TC, pH 6.0–6.3, 800 mL) and descending (DC, pH 6.4–6.9, 600 mL), as described in Firmann et al. [76]. The colonic vessels for both lines were inoculated with a fresh fecal sample from a healthy subject who agreed to participate in the study by signing a written consent for all procedures and surveys, which were previously approved by the local ethical committee of the Institute of Nutrition and Food Technology of the University of Chile (code PT8-2022) and conducted in accordance with the principles outlined in the Declaration of Helsinki and the International Ethical Guidelines for Health-related Research Involving Humans (2009). Informed consent was obtained prior to their inclusion in the study. The participant reported regular adherence to a Mediterranean-style dietary pattern and had not received antibiotics for 3 years prior to sample collection. Two weeks after inoculation were considered for stabilization of the microbiota, and during this period the system was fed daily every eight hours (01.00, 09.00, 17.00h) with 140 mL culture media (Cat. No. PD-NM002B, Prodigest, Belgium), in g/L: 1 arabinogalactan, 2 pectin, 1 xylan, 3 potato starch, 0.4 glucose, 3 yeast extract, 1 pepton, 4 mucin, and 0.5 cysteine. It is important to note that the SHIME system enables real-time observation of acidification in colonic bioreactors during each feeding cycle. When the microbiota becomes stable, acidification steps become regular. Additionally, SCFA levels measured over the following 4 days of baseline indicate fluctuations of 6–12% for all four SCFAs (acetate, propionate, butyrate, and valerate) across the three colon compartments [24 measurements for each SCFA member]. After baseline, 4g of NDF-g was incorporated in one of the lines as the phenolic-rich fiber. In the control line, an equivalent mass of NDF-g was subjected to a double-exhaustive extraction procedure to deplete its phenolic content. To eliminate any trace of organic solvents, this fiber was profusely rinsed with distilled water and dried at 60 °C for twelve hours. This phenolic-depleted fiber was incorporated into the control line (see the Figure 4).

The 4 g of NDF-g was obtained starting from 20 g of skin grape pomace subjected to oral–gastric digestion, which, after a centrifugation step (3000 g, 15 min) and drying at 60 °C, rendered that amount of insoluble non-digestible fraction. This was incorporated into the SHIME system at the level of the small intestine. It is noteworthy that 20 g of skin grape pomace contains the equivalent amount of phenolic compounds as that consumed daily, according to the 24-h recall EPIC survey of 4000 European subjects [77]. During the whole study, samples were collected daily at fixed time points (11.00 h) from the three colon vessels, centrifuged at 5000 g for 2 min at 4 °C, and stored at −80 °C until further analysis.

### 4.6. Short-Chain Fatty Acid Extraction and Determination by Gas Chromatography

Ten microliters of 6 mM tropic acid (Internal standard, Cat. No. T89206, Sigma-Aldrich, USA) were mixed in 100 mL of samples from colonic reactors, and 1mL of fuming HCl was added. To this mixture, 200 mL diethyl ether was then added, vortexed for 30 min, and decanted for 2 min to allow the separation of aqueous and organic phases; the upper phase (organic) was collected in a new tube, and this extraction was repeated three more times, collecting the organic phase along with the previous one. A pinch of NaSO_4_ was added to remove any possible traces of water. To proceed with the SCFA derivatizing, 10 µL of N,O-Bis(trimethylsilyl)trifluoroacetamide (BSTFA, Cat. No. 15222, Merck, Germany) was added to 200 µL dehydrated organic phase in a glass vial and incubated at 37 °C for 1 h. One microliter from the glass vial was injected into the Gas Chromatography Mass Spectrometry equipment (GCMSD 7890A/5975, Agilent, Santa Clara, CA, USA), which was coupled with a mass spectrometer detector and supported with a fused silica column Rxi-5ms (30m, 0.25mm ID, 0.25 µm, Restek, Bellefonte, PA, USA). The separation of SCFAs was carried out with a flow rate for helium of 1mL/min and a temperature ramp that initiated at 40 °C, maintained for 2 min, raised to 150 °C at 15 °C/minute, held for 1 min, increased to 300 °C at 30 °C/minute, and finally held to 300 °C for 5 min. The total run time was 20 min. The inlet and auxiliary temperatures were 260 °C and 280 °C, respectively. Another microliter from the derivatized sample was injected in split mode (1:25). Solvent delay was set to 3 min, and mass detection was obtained using full-scan mode in the range of 50- 480 m/z with an ionization energy in electro impact mode of 70 mV. Compound identification was validated by injecting pure standards and comparing retention times and corresponding MS spectra. Selected ion monitoring (SIM) mode was used for quantification, utilizing the target ion, and confirming with qualitative ions. Specifically, the target ions (m/z) for acetic, propionic, butyric, valeric, and tropic acids were 117, 131, 145, 159, and 280, respectively. The quantification of the abundance (micromoles) of SCFAs in every independent sample was achieved by interpolating their relative abundance on a freshly prepared calibration curve with the SCFA standards, purchased from RESTEK, USA (Cat. No. 35272), and the ChemStation MSD Data Analysis software from Agilent, USA.

### 4.7. Total Phenolic Content (TPC)

TPC was determined using the Folin–Ciocalteu assay with modifications. Briefly, samples were thawed and stirred, then diluted to an appropriate concentration relative to gallic acid. Briefly, 100 μL of sample, 200 μL of 10% (*v*/*v*) Folin–Ciocalteu reagent, and 800 μL of 0.7 mM sodium carbonate were mixed, and the solution was left to stand for 120 min for the reaction to take place and stabilize. Absorbance was measured at 765 nm. Gallic acid was employed as a calibration standard, and results were expressed as gallic acid equivalents (mg GAE/g extract).

### 4.8. Identification and Quantification of Phenolic Compounds

Quantification of phenolic compounds present in extracts and ferments was performed using HPLC equipped with a photodiode array detector [200–550 nm] and Chromaster System Manager V1.2 as analysis software (Hitachi Chromaster 5000 series, Tokyo, Japan) following the method described by Bridi et al. [78]. A total of 10 µL of the sample was injected into an RP-18 Purospher STAR column (250 × 4.6 mm i.d.). The elution was carried out using a gradient of three mobile phases at 35 °C: (A) methanol, (B) acetonitrile, and (C) formic acid (0.1%). The gradient program for chlorogenic, syringic, caffeic, ferulic, synapic, abscisic, trans-cinnamic, and coumaric acids and catechin, rutin, daidzein, quercetin, naringenin, genistein, apigenin, kaempferol, isorhamnetin, pinocembrin, and chrysin y galangin was: 0–10 min 20% B, 80% C; 10.1–40 min 7.5% A, 25% B, 67.5% C; 40.1–50 min 15% A, 25% B, 60% C; 50.1–65 min 15% A, 45% B, 40% C; 65.1–80 min 20% B; and 80% C, at 0.8 mL/min. Additionally, the acids, gallic, protocatechuic and vanillic, epicatechin, procyanidin B1 and B2, kaempferol 3-rutinoside, isoquercitrin, taxifolin, epigallocatechin, and epicatechin gallates were separated with the following gradient program: 0–30 min (15% A, 85% B), 30.1–45 min (25% B, 75% C), 45.1–55 min (40% B, 60% C), 55.1–60 min (50% B, 50% C), 60.1–65 min (80% B, 20% C), and 65.1–75 min (15% B, 85% C) at 1 mL/min. A diode array detector (DAD) was used to measure the absorbance of the eluate (210–700 nm), and the chromatograms were integrated for both standards and extracts at 290 nm. Retention times of the standards and the UV-vis spectra were used for the identification. For quantification, a multi-standard combination was also performed in equal concentrations of each compound (range 5–200 μM) to obtain calibration curves of all standards studied [79]. All measurements were performed in triplicate, and results were expressed as micrograms per gram of dry sample (µg/g).

### 4.9. Caco-2 Cell Differentiation and Treatment with Ferments

Human colon adenocarcinoma cells (Caco-2, ATCC HTB-37 TM) were acquired from the American Type Culture Collection (Manassas, VI, USA). Cells were cultured in Minimal Essential Medium supplemented with fetal bovine serum (10%) and penicillin/streptomycin (1%). The cultures were maintained at 37 °C in a humid atmosphere with 5% CO_2_ and 95% air. At passage 14, cells were seeded onto 0.4 µm pore-size polycarbonate Transwells (Corning, NY, USA), and the medium was refreshed twice weekly. Transepithelial electrical resistance (TEER) was monitored every 2–3 days with an EVOM2 device (Hertfordshire, UK). By the third week, the TEER values of the Caco-2 monolayer were stable and above 700 Ω/cm^2^.

SHIME ferments were mixed with culture medium at equal proportions to be added at the apical side of differentiated Caco-2 cells. TEER values were continuously monitored for up to 7 h of exposure. After this period, apical media were replaced with full-supplemented culture medium. The next day, a second SHIME ferment exposure followed the same procedures described above. After the second resting period, total RNA from monolayers was isolated for RNA sequencing analysis.

### 4.10. RNA Isolation

Each basquet Transwell with approximately 1.5 × 10^5^ Caco-2 cells was lysed with TRIZOL (Cat. No. 15596018, ThermoFisher, Waltham, MA, USA). The quantity and purity of total RNA from the samples were estimated by measuring the absorbance (ratio 260/280 nm) in the spectrometer NanoDrop 2000 (Thermo Scientific Technologies, Waltham, MA, USA). RNA integrity (RIN) was evaluated using the Agilent 2200 TapeStation (Agilent Technologies, Inc., USA). For RNA-seq, only samples with high-quality RNA 260/280 ≥ 1.8 and RIN ≥ 8.5 were considered.

### 4.11. RNA Sequencing and Bioinformatic Analysis

Messenger RNA (mRNA) sequences were enriched for the preparation of libraries with the Dynabeads mRNA Purification Kit. Libraries were generated with the MGIEasy RNA Library Prep Kit and the MGIEasy Circularization Module V2.0. Sequencing was carried out in the paired-end modality (2 × 150 pb) using the DNBSEQ-G400RS High-throughput Sequencing Set kit (MGI) in a DNBSEQ-G400 equipment, which rendered between 18.7 and 68.9 million reads per sample. In order to check the quality of raw FASTQ files, the software FastQC (v0.12.1) was employed. Trim Galore v0.6.10 allowed the selection of high-quality reads with a mínimum Phred score of 30 and length over 75 bp. The alignment of sequences with the reference genome for *Homo sapiens* (GRCh38.p14, NCBI) was carried out with STAR v2.5.2b [80]. The bioinformatic analysis proceeded to compare the differential expression between two conditions: Caco-2 cells exposed to colonic ferments in the presence of FNDg and exposed to a control fiber, in which the FNDg was depleted of phenolic compounds. Differential expression analysis was performed using two distinct statistical frameworks: limma–voom(v3.62.2) [81,82] and DESeq2 (v1.46.0) [83]. For the limma–voom pipeline, raw counts were normalized using the TMM (Trimmed Mean of M-values) method to correct for differences in library size. The voom transformation was applied to convert counts to log2 counts-per-million (logCPM), and gene-wise linear models were then fitted using Empirical Bayes moderation and moderated t-statistics. In parallel, the default DESeq2 pipeline was employed, using a Negative Binomial Generalized Linear Model (GLM) fitting and the Wald test for statistical inference. For both pipelines, genes were defined as significantly differentially expressed (DEGs) if they exhibited an expression level twice that of the other condition (|log2 fold change| > 1) and a *p*-value < 0.05, adjusted by the Benjamini–Hochberg method.

Functional enrichment analysis was performed using clusterProfiler (v4.14.6) [84], from a list of significant up-regulated DEGs identified by limma. Enrichment analysis was conducted for Gene Ontology (GO) categories, including Biological Process (BP), Molecular Function (MF), and Cellular Component (CC), as well as Kyoto Encyclopedia of Genes and Genomes (KEGG) pathways. Terms with a Benjamini–Hochberg adjusted *p*-value < 0.05 were considered significantly enriched. The RNA sequence database is available at the GEO repository under accession GSE317808 (NCBI, USA).

### 4.12. Statistical Analysis

All data presented in this work are expressed as the average ± standard error from at least three independent experiments. When two or more conditions were compared, an ANOVA test with Bonferroni post hoc was executed. For non-parametric data, the Kruskal–Wallis test with Dunn’s post hoc test was used. Plots and data analysis were carried out with Sigma Plot 12.0 (Systat Software, Inc., San Jose, CA, USA, EE. UU.). It is important to note that in this study, colonic fermentation was performed using a single sample from a healthy subject. For bioassays on Caco-2 cells, experiments were performed in triplicate, and RNA-seq analysis was conducted using the two best biological replicates.

## 5. Conclusions

- Digestion and colonic fermentation facilitate the release of certain phenolic compounds, highlighting the colonic release of catechins.
- Differentiated Caco-2 cells survive repeated exposure to colonic ferments.
- The exposure to phenolic-rich colonic ferments modifies the transcriptomic profile of differentiated Caco-2 cells, which is critical for cell-to-cell communication and the function of the apical domain.

## Figures and Tables

**Table 1:** Quantification of individual phenolic compounds (µg/g dw) in skin grape pomace before (undigested samples) and after in vitro gastric digestion, intestinal digestion, and ascending colon fermentation, determined by HPLC-DAD.

	Skin Grape Pomace					
	Undigested	Gastric Digestion		Intestinal Digestion		Colon Fermentation
		Digestible	Non-Digestible	Digestible	Non-Digestible	
Phenolic Acids:						
Abscisic acid	nd	nd	nd	nd	50 ± 3	nd
Caffeic acid	nd	nd	nd	nd	d	nd
p-Coumaric acid	nd	nd	nd	nd	d	nd
Syringic acid	325 ± 28	nd	d	32 ± 2	471 ± 11	nd
Sinapic acid	1062 ± 67	nd	nd	nd	152 ± 10	nd
trans-Cinnamic acid	nd	nd	nd	nd	d	nd
trans-ferulic	nd	nd	nd	nd	nd	d
Gallic acid	d	nd	nd	nd	nd	nd
Flavones:						
Apigenin	nd	nd	nd	nd	d	nd
Flavonols:						
Galangin	nd	nd	nd	nd	d	nd
Kaempferol	nd	d	267 ± 3	nd	116 ± 1	nd
Isoquercitrin	nd	nd	nd	nd	nd	nd
Quercetin	nd	nd	nd	nd	51 ± 1	nd
Rutin trihydrate	nd	nd	nd	nd	d	45 ± 4
Flavan-3-ols:						
Catechin	nd	nd	nd	nd	nd	188 ± 2
Epicatechin	nd	d	nd	nd	nd	6 ± 0.4
Epigallocatechin gallate	nd	nd	nd	nd	21 ± 0.4	nd
Epicatechin gallate	nd	nd	nd	nd	d	nd
Procyanidin B2	nd	nd	nd	nd	nd	nd
Flavanones:						
Pinocembrin	nd	nd	nd	nd	d	nd
Taxifolin	nd	nd	131 ± 1	nd	nd	nd

Results are expressed in µg per gram of dry extract weight (µg/g dw) and presented as mean ± SD (*n* = 3). ND: not detected. D: detected below the lowest concentration used in the calibration curve.

## Data Availability

The original contributions presented in this study are included in the article/Supplementary Material. Further inquiries can be directed to the corresponding author(s). Data from RNA-seg is available at the GEO repository under accession GSE317808 (NCBI, USA).

## Supplementary Materials

The following supporting information can be downloaded at: https://www.mdpi.com/article/10.3390/ijms27094123/s1.
