# Supplementary material for: Phenolic Compounds of Grape Pomace Skin Released During SHIME Colonic Fermentation Shape the Transcriptomic Profile of Tight Junctions, Improving the Barrier Properties in Caco-2 Cells
Source: Int J Mol Sci. 2026 May 5;27(9):4123. doi: 10.3390/ijms27094123 (PMC13163868; doi:10.3390/ijms27094123)
Supplement: Supplementary file 1 [file ijms-27-04123-s001.zip › ijms-4245807-supplementary.pdf]

Supplemental Figure S1.

Grape pomace treatments did not increase short-chain fatty acid levels in the ascending, transverse, or descending colon reactors of the TWINSHIME.

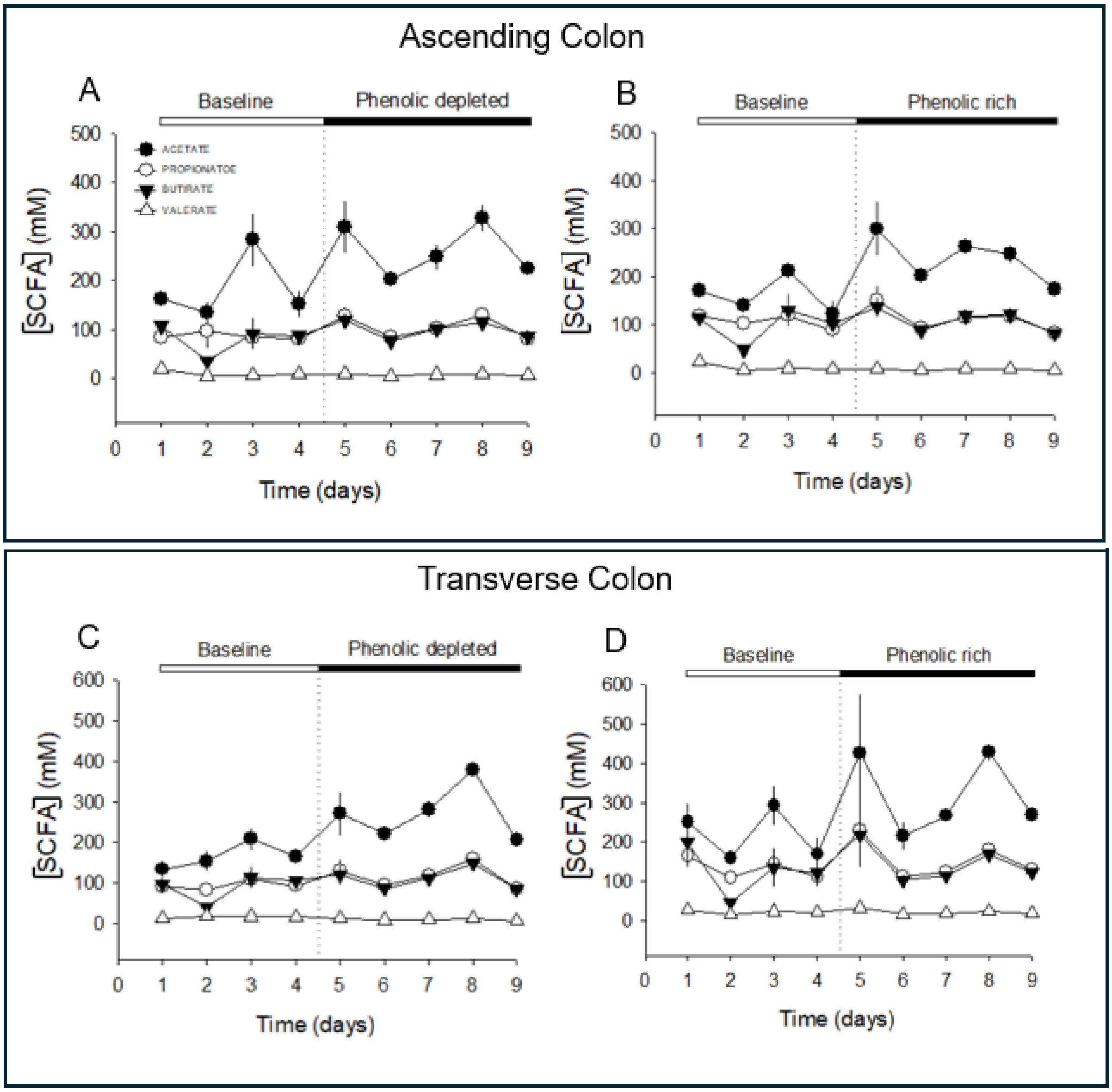

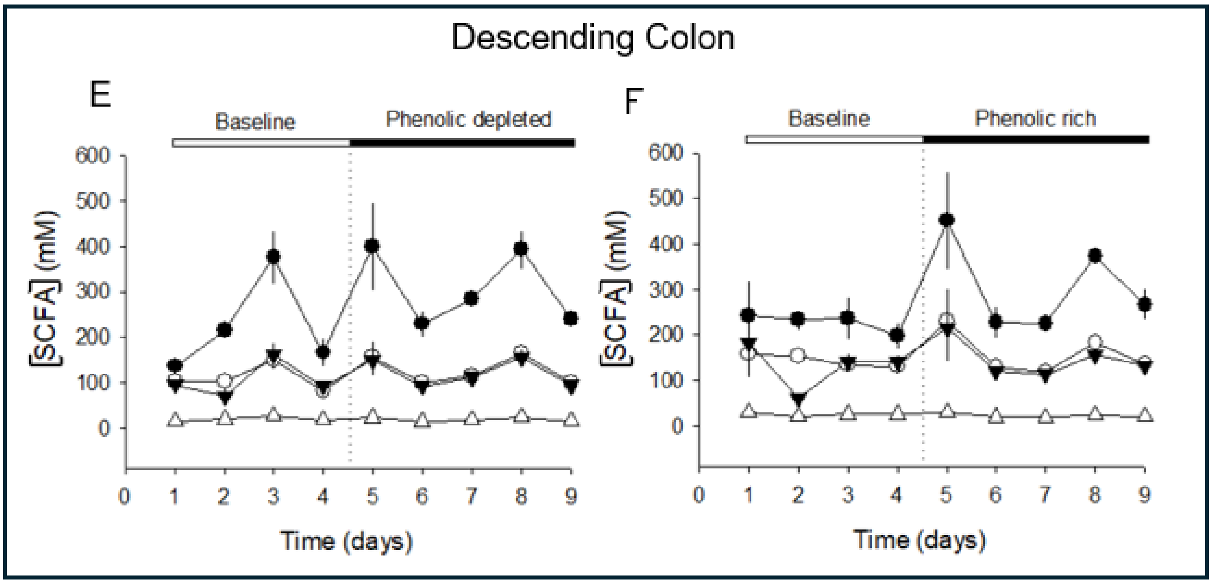

Comparison of the time courses observed for acetate (filled circles), propionate (empty circles), butyrate (filled triangles) and valerate (empty triangles) levels in the ascending (upper panel), transverse (middle panel) and descending (lower panel) colonic vessels. Each course shows a baseline period (white bar) followed by the treatment with phenolic-depleted fiber (A, C and E) or phenolic-rich fiber (B, D and F). Data correspond to the mean ± ES (n=3). Statistical analysis was performed using repeated-measures ANOVA. No statistically significant differences were found for any of the short-chain fatty acids measured in any colonic bioreactor.

Supplemental Figure S2.

Monitoring the Formation of a Caco-2 Cell Monolayer Through the Measurement of Transepithelial Electrical Resistance (TEER).

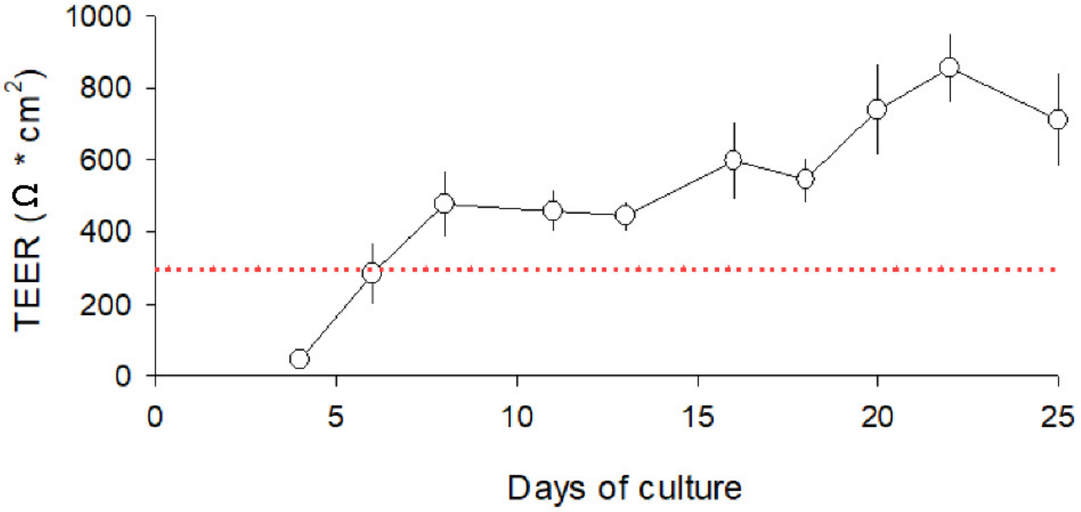

On day 1, 300,000 cells were seeded on bicameral inserts and transepithelial electrical resistance (TEER) was measured every 3 or 4 days. The red dashed line indicates 300 Ω*cm², which represents an intact monolayer.

Each TEER value represents the mean ± SEM from 9 biological samples from 2 independent experimental assays.

Supplemental Figure S3.

Principal Component Analysis (PCA) of global gene expression in Control and Treatment samples.

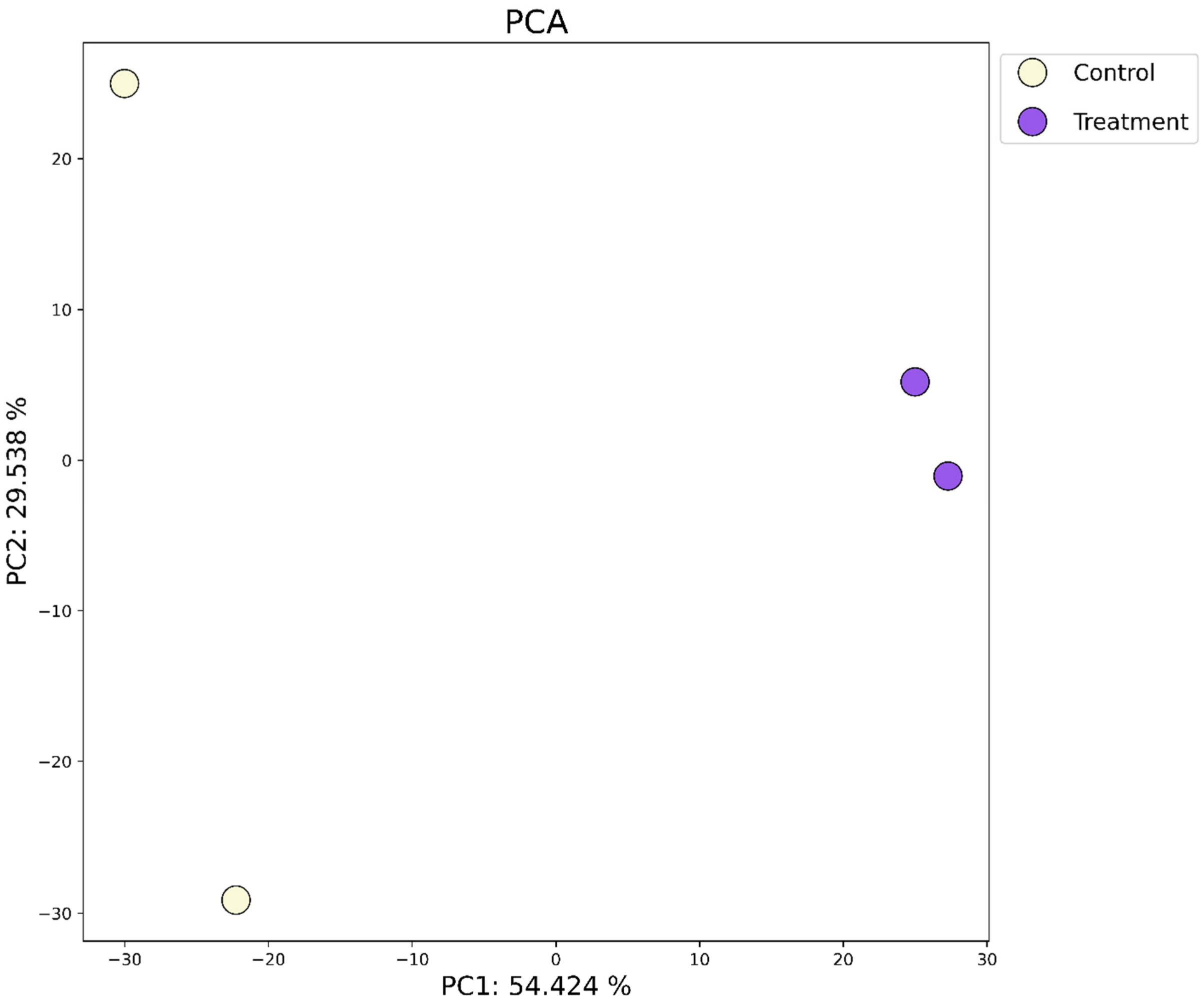

The score plot illustrates the distribution of samples along the first two principal components. PC1 and PC2 account for 54.42% and 29.54% of the total variance, respectively. Individual points represent Control (yellow) and Treatment (purple) replicates (per group), positioned according to their coordinate scores.
